# Action Sounds Modulate Arm Reaching Movements

**DOI:** 10.3389/fpsyg.2016.01391

**Published:** 2016-09-16

**Authors:** Ana Tajadura-Jiménez, Torsten Marquardt, David Swapp, Norimichi Kitagawa, Nadia Bianchi-Berthouze

**Affiliations:** ^1^UCL Interaction Centre, University College LondonLondon, UK; ^2^Department of Psychology, Universidad Loyola AndalucíaSeville, Spain; ^3^UCL Ear Institute, University College LondonLondon, UK; ^4^Department of Computer Science, University College LondonLondon, UK; ^5^NTT Communication Science Laboratories, NTT CorporationKanagawa, Japan

**Keywords:** auditory-dependent body-representation, action sounds, body-related sensory inputs, body kinematics, goal directed actions

## Abstract

Our mental representations of our body are continuously updated through multisensory bodily feedback as we move and interact with our environment. Although it is often assumed that these internal models of body-representation are used to successfully act upon the environment, only a few studies have actually looked at how body-representation changes influence goal-directed actions, and none have looked at this in relation to body-representation changes induced by sound. The present work examines this question for the first time. Participants reached for a target object before and after adaptation periods during which the sounds produced by their hand tapping a surface were spatially manipulated to induce a representation of an elongated arm. After adaptation, participants’ reaching movements were performed in a way consistent with having a longer arm, in that their reaching velocities were reduced. These kinematic changes suggest auditory-driven recalibration of the somatosensory representation of the arm morphology. These results provide support to the hypothesis that one’s represented body size is used as a perceptual ruler to measure objects’ distances and to accordingly guide bodily actions.

## Introduction

In our everyday interaction with the environment, most of us perform many physical actions that allow our body to reach, grab or point at different objects. For most of us these actions seem to occur smoothly, mostly automatically. This successful and smooth interaction with the environment is enabled by the use of internal models of body shape and posture (Head and Holmes, 1911–1912; Maravita and Iriki, 2004). To account for changes in body configuration or structural changes that occur when the body moves or ages, these body models are continuously updated (Sirigu et al., 1991). Neuroscience and psychological research have repeatedly shown that updates in the internally represented body models, the so-called body-representations, occur in response to the continuous multisensory input we receive on our body (Tsakiris, 2010). For instance, experiments on the “rubber-hand illusion” (RHI) demonstrated that a rubber arm can be incorporated into one’s body model, if one observes touches to the rubber arm while synchronously feeling touch delivered to their own, unseen, arm (Botvinick and Cohen, 1998). This illusion results from the integration of congruent sensory information received through vision and touch. Other studies have shown that feelings of ownership over a rubber hand can be also elicited during active-touch conditions, in which one delivers touch to the seen fake hand and in synchrony receives touch to one’s own unseen hand (Aimola Davies et al., 2010), and by synchronous seen and felt movement of a hand and one’s own unseen hand (Tsakiris et al., 2006; Newport et al., 2010; Sánchez-Vives et al., 2010; Kalckert and Ehrsson, 2012), thus highlighting the influence of proprioceptive cues in this illusion.

Similarly, previous studies have shown that wielding a tool that serves to act with one’s arm upon relatively distant objects, in other words, a tool that physically extends the arm, yields an extension of the represented arm length (Cardinali et al., 2009, 2012; Canzoneri et al., 2013b). In this case, extension of the represented arm length also derives from the integration of congruent sensory information, as visual events at the tip of the tool are contingent with tactile information received at the hand (Heed and Röder, 2012). At the same time, updates in body-representation can occur without the involvement of vision. For instance, some studies have shown that vibrations delivered to one’s bicep tendon or wrist may induce the illusory feeling of one’s arm extending thus altering the perceived position of one’s hand in space. When this hand is touching another body part (e.g., a finger of the other hand, one’s waist) one may experience distortions in the represented shape or size of this other body part (Lackner, 1988; de Vignemont et al., 2005; Ehrsson et al., 2005).

More recently it has also been demonstrated that updates on body-representation can occur through audition, thus providing evidence of the supramodal nature of body-representation (Azañón et al., 2016 for a review). For instance, altering the sound of the impact of an object on one’s hand modifies the felt material of one’s own hand (Senna et al., 2014). Apart from these effects of sound in perceived body material properties, in a previous study we showed that altering action related sounds can elicit changes in the represented body dimensions. In particular, we showed that altering the perceived position of the sounds produced by one’s hand when tapping on a surface recalibrates the represented length of one’s arm (Tajadura-Jiménez et al., 2012). These changes in represented length of one’s arm were evidenced by changes in perceived tactile distances on the arm (a measure also used by Taylor-Clarke et al., 2004; de Vignemont et al., 2005; Canzoneri et al., 2013a,b; Miller et al., 2014). A subsequent study showed that the observed changes in perceived tactile distance correlated with feelings of one’s arm having elongated (Tajadura-Jiménez et al., 2015b).

Because the representation of an action engages a mental representation of the general body structure that allows this action to be produced (Holmes and Spence, 2004; Maravita and Iriki, 2004), it is often assumed that the changes in body-representation evoked by multisensory bodily inputs will have implications in the way actions are performed. However, only a few studies have actually looked at the effects of these body-representation changes on movement or goal directed actions. Among these studies are those reporting that the RHI influences subsequent grasping responses, as a consequence of the grip aperture of the rubber hand (Kammers et al., 2010) or the visual size of the rubber hand (Marino et al., 2010; see also related effects on perceived weight of objects reported by Haggard and Jundi, 2009), or the studies showing that the RHI influences subsequent reaching movements, as a consequence of the shift in the perceived hand position (Newport et al., 2010; Zopf et al., 2011), although note that Kammers et al. (2009) failed to find such an influence on reaching movements. The majority of studies have rather looked at changes in bodily feelings, perceived position of the body in space or perceived tactile distances, as reviewed above. Some of these changes might be linked to updates in internal models that are aimed to facilitate successful and smooth interactions with the environment. For instance, changes in tactile distance perception suggest recalibration of somatosensory receptive fields (Tajadura-Jiménez et al., 2012), and the control of body movements is known to rely on the somatosensory representation of the body morphology (Holmes and Spence, 2004; Maravita and Iriki, 2004). Indeed, Cardinali et al. (2009) showed that using a 40 cm-long mechanical grabber resulted both in shifts in tactile localization and in alterations in the kinematics of subsequent reaching movements performed without the tool: these movements changed in a way consistent with reaching movements performed with a ‘longer’ arm. However, given that multiple body-representations coexist in the human brain and that their plasticity is a task dependent process (Longo and Serino, 2012), the question of whether action sounds can evoke changes in the internal models of arm morphology involved in facilitating action remains open.

In the present study we investigated the potential effect of action sounds on subsequent goal directed actions. In particular, we took measures of arm kinematics before and after exposure to the audio-tactile adaptation phase used in our previous studies to induce alterations in the represented arm length (Tajadura-Jiménez et al., 2012, 2015b). We adopted the reaching task from Cardinali et al.’s (2009) study in which the reaching movements after a period of tool-use were characterized by longer latencies and reduced amplitudes in velocity and acceleration movement parameters. As the authors showed in an additional experiment, these changes in kinematics are consistent with reaching movements performed with a ‘longer’ arm, as the very same kinematic differences were found when comparing free-hand grasping movements of individuals with a longer arm with those movements performed by individuals with a shorter arm. By looking at whether people *behave* as if their arm was longer, we aim to provide a measure of implicit changes in the represented arm that relates to goal directed actions.

## Materials and Methods

### Participants

Eighteen participants (*M*_age_ ±*SD* = 22.61 ± 4.1 years; age range from 18 to 32 years; nine females and nine males) took part in the experiment. All participants reported having normal hearing and normal tactile perception, and were naïve as to the purposes of the study. Behavioral data from one of the conditions for two participants were lost due to recording problems, and therefore, all data from these two participants were excluded from the analyses, which were performed on the remaining sixteen participants (*M*_age_ ±*SD* = 23 ± 4.2 years; age range from 18 to 32 years; eight females and eight males). Arm length was not taken into account when recruiting participants but there was a reasonable variation in arm length across participants (*M* = 71.69 cm, *SD* = 6.02 cm, range 63–81 cm). Participants were paid for their time and gave their informed written consent prior to their inclusion in the studies. The experiment was conducted in accordance with the ethical standards laid down in the 1964 Declaration of Helsinki and approved by the ethics committee of University College London.

### Apparatus and Materials

**FIGURE 1:**
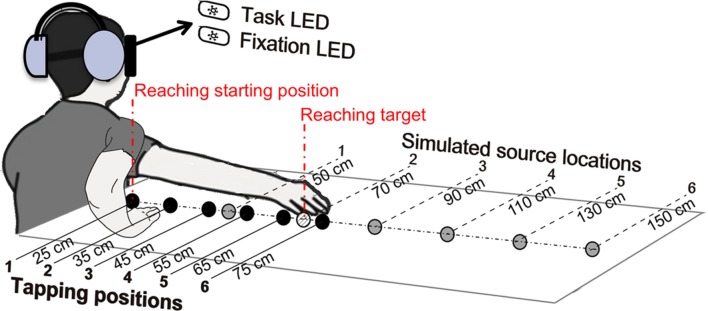
**Experimental set-up.** The two illustrations of the right arm displayed correspond to the positions adopted at the beginning and at the end of the audio-tactile “tapping” task. During this task participants started tapping at the first tapping-position, with the arm flexed, and then progressively moved their hand along the six tapping-positions, represented in black color, ending with the arm extended. Participants heard the taps via the headphones as originating from the simulated source locations, represented in gray color. The target object in the reaching task was placed approximately at the same location as the second simulated source (see Materials and Methods). Figure adapted from Tajadura-Jiménez et al. (2015b).

The apparatus and materials used for the audio-tactile “tapping” task (see the next section) were identical to those used in Tajadura-Jiménez et al. (2015b). A schema of the experimental set-up is displayed in **Figure 1**. Participants were seated in a chair, blindfolded and wearing a pair of closed headphones with very high passive ambient noise attenuation (Sennheiser HDA 200). A table was placed to the right of the participants. The height between the participants’ right ear and the surface of the table was approximately 40 cm. A pair of light-emitting diodes (LEDs) of different colors was positioned in front of the participants, at eye level and a distance of 50 cm. They were bright enough so that participants could see the light through the blindfold. The ‘fixation’ LED served as the center fixation point, and the ‘task’ LED was used by participants to perform the experimental task, as described in the next section. During the experimental sessions participants were instructed to refrain from turning their head sideways from the fixation point.

In **Figure 1**, one can see an array of six “tapping-positions” (marked in black color), and an array of six simulated “source locations” (marked in gray color), which replicate those used in our previous study (Tajadura-Jiménez et al., 2015b) and that proved to be effective in eliciting changes in the represented arm length. The participants were instructed to tap on the surface of the table, at the six different “tapping-positions,” which were located 90° to the right, respectively, at 25, 35, 45, 55, 65, and 75 cm from a vertical line traced between the participants’ right ear and the table surface. We simulated the auditory “source locations” by using virtual acoustic techniques with the procedure described in Tajadura-Jiménez et al. (2015b). This simulation used a “dry” pre-recorded sound of two fingers tapping on a cardboard box (125-ms duration and broad spectrum) and modified it to provide the listener with distance and directional cues so that the “source locations” were in the direction of the tapping-positions but at double their distances; thus, the distance between two tapping-positions was doubled (i.e., if the distance between two tapping-positions was 10 cm, the simulated distance between two virtual source locations was 20 cm). This resulted in the sixth source location in the array to be located 150 cm away from the vertical line traced from the participants’ ear (while the actual tapping-position was 75 cm away). An additional set of virtual source locations (“practice array”) was identical to the actual tapping-positions and was used in the practice session that participants performed to get familiar with the tasks. Please refer to our previous article (Tajadura-Jiménez et al., 2015b) for more details on the simulation of the virtual auditory source locations.

A piezoelectric transducer (Schaller Oyster 723 Piezo Transducer Pickup), attached to the table, was used to detect the participants’ taps and trigger the auditory stimulation. In the Synchronous condition, the auditory stimulus was triggered in synchrony with the participant’s tap on the table. To note, the latency of the signal-processing module was in total less than 4 ms. Such short latency is unperceivable across sensory modalities, as it falls well within the intersensory temporal synchrony window (Lewkowicz, 1996, 1999). In the Asynchronous condition, the auditory stimulus was triggered with a small delay with respect to the participant’s tap. This delay varied randomly over a range of 300–800 ms. The minimum delay value of 300 ms was chosen to fall outside of the multisensory integration window during which asynchronous stimuli in different modalities are perceived as simultaneous (Lewkowicz, 1996, 1999). MATLAB software was used to control a real-time sound processor RP2 (Tucker-Davis Technology) for the stimulus delivery and to record the participant’s responses.

The actual sound of the participants’ taps on the table was attenuated by the high ambient-noise attenuation headphones, and masked by adding a low level of background noise (interaural uncorrelated pink noise, 20–13000 Hz) to the headphone signals throughout the entire duration of the audio-tactile ^..^ tapping ^..^ task (see Procedure section).

The apparatus used for the kinematic recordings consisted of four reflective markers placed on the participant’s right arm. The first marker was placed on the dorsum of the distal phalanx of the index finger; the second marker was placed on the styloid process of the radius at the level of the wrist; the third marker was placed on the elbow joint on the outer side; and the fourth marker was placed on the shoulder joint on the scapular acromion. An additional marker constituted the target object. The spatial position of the markers was recorded with an optical motion capture system (Vicon V-series comprising six infrared cameras) with a sampling rate of 100 Hz and less than 3 mm 3D resolution at an applied distance of approximately 5 m. In this experiment the 3D resolution was even higher than 3 mm since the applied distance was smaller than 2 m.

### Tasks

#### Reaching Task

At the beginning of the experimental session, blindfolded participants were instructed to search for a target object with their hand and to touch it so to memorize its location (Abekawa and Gomi, 2010). This target object was placed midway between the fifth and the sixth tapping-positions, thus at a distance of 45 cm from the first- tapping position. Then, before performing the “reaching” task, blindfolded participants were instructed to wait for a go-signal (a 500 Hz tone, lasting 250 ms) while resting their right index finger in the first tapping-position (starting position). Upon hearing the go-signal, they were asked to perform as rapidly and accurately as possible the action of reaching and touching with their same index finger the target object (as in Cardinali et al., 2009). A piece of Velcro on the top of the first tapping-position helped participants to find this position by touching. Participants were asked to keep their hand in the air while reaching to the target. During the experiment, the experimenter kept close to participants and visually monitored that the reaching movements were performed as required.

Note that when performing this reaching task, since participants were blindfolded, they were not always correct in reaching the target when completing their movement. If participants failed to reach the target during a reaching trial, they were subsequently asked to search for the target by means of tactile exploration, so that they confirmed its location before the start of next adaptation phase (as in Abekawa and Gomi, 2010). These trials were not excluded from the analysis as failures to reach the target served as a measure of recalibration of the length of the represented arm.

The reaching task was repeated twelve times before the first audio-tactile tapping task (Pre-test) and then once more after each audio-tactile tapping task (Post-test). Pre-test served as a baseline measure to which to refer the post-test values (post-adaptation measure). The instructions and task were identical for the Pre-test and the Post-test in all respects, including the instruction to perform a tactile exploration until reaching the target, before moving on to the next adaptation phase, in case of failure in reaching it during the first attempt.

#### Audio-Tactile “Tapping” Task (Adaptation Task)

As displayed in **Figure 1**, participants were required to look straight to the ‘fixation’ LED and to perform the simple action of tapping on the table using their right hand, while keeping their palm open and their arm ventral side down [see Tajadura-Jiménez et al. (2012, 2015b) for similar procedures]. They tapped with four of their fingers (index to small finger), starting at the first tapping-position (closest to their body), 10 times and the auditory stimulus was delivered at the first source location in the array, in synchrony or in asynchrony with the participant’s tapping, depending on the condition. Participants were asked to pace their rhythm keeping a frequency of approximately one tap per second.

After 10 taps, a signal (‘task’ LED) indicated to the participants to extend their arm rightward by 10 cm, and tap again 10 times at the new tapping-position (farther from their body), with the auditory stimulus presented from the subsequent source location in the array (i.e., at double the distance to the tapping-position). This procedure was repeated for all six tapping positions, making a total of 60 taps on the table and ending with the farthest away tapping position and simulated source location. The Asynchronous condition served as the control condition as asynchrony disrupts the feelings of agency over the tapping sounds and these feelings are necessary in order for auditory inputs to change body-representation (Tajadura-Jiménez et al., 2012, 2015b).

After completion of the 60 taps conforming the tapping task participants were instructed to move their arm back to the initial starting position and wait for a go-signal to perform the reaching task.

### Procedure

At the start of the experimental session, the participant’s arm length was measured from the right acromion (shoulder joint) to the middle finger-tip in order to check for possible individual differences in arm kinematics due to arm length (see Longo and Lourenco, 2007; Cardinali et al., 2009, for similar procedures). After receiving instructions, participants were then asked to practice all the tasks. For the tapping task, they were encouraged to keep close to the location of the six tapping-positions. Participants first practiced without wearing the blindfold, and then, once again wearing the blindfold, with the experimenter giving them feedback on their performance. The audio-tactile “tapping” task in this practice block differed from the one in the experimental blocks in that the “practice array” of source locations was used. Although the tapping-positions were not marked in such a way that participants could feel them by touching (except for the first position that had a piece of Velcro on the top, as previously mentioned), it was expected that the extensive practice before the experiment start would lead participants to tap approximately at the tapping-positions. In any case, the experimenter kept close to participants and visually monitored that the required pace and distances of movement were kept during the whole experiment, and when necessary (only on a couple of occasions), corrected participants by grabbing and leading their hand to the exact tapping-position.

Next, participants completed two experimental sessions, each containing two stages: (1) 12 repeats of the pre-stimulation reaching task (Pre-test), (2) 12 repeats of an experimental trial in which participants first performed the audio-tactile tapping task (60 taps, 10 taps in each tapping-position) and immediately after this adaptation phase they performed a post-stimulation reaching task (Post-test). Participants were blindfolded throughout the experimental session. The experimental sessions differed in the auditory condition (Synchronous or Asynchronous) during the audio-tactile tapping task. Each experimental session (Synchronous or Asynchronous) lasted on average for 20 min. The order of their presentation was randomized.

At the end of each session (Synchronous or Asynchronous), the subjective experience of participants during the audio-tactile tapping task was assessed with a questionnaire containing eight statements, adopted from our previous studies (Tajadura-Jiménez et al., 2012, 2015b). The list of statements is presented in the Results section. The questionnaire statements assessed whether participants felt they had caused the sound they heard, whether they felt their hand was at the same location as the sound or couldn’t really tell where their hand was, whether they felt their arm longer or shorter than usual or couldn’t remember its length, whether they felt their arm was out of their control or whether they had a less vivid feeling of their arm. Participants rated their level of agreement with the statements using a 7-item Likert scale, ranging from -3 (strongly disagree) to +3 (strongly agree), with 0 referring to “neither agree, nor disagree.” Based on our previous findings, we expected the asynchrony condition to disrupt the feelings of agency over the sound that was caused by their action and of spatial congruency between hand and sound, and that it would result in larger feelings of losing control over the arm (Tajadura-Jiménez et al., 2012, 2015b). Larger level of agreement with the statement on feeling of arm elongation would provide evidence of the expected illusion due to the audio-tactile stimulation, while we did not expect the feeling of one’s arm being shorter. The rest of the statements were included to further enquire whether exposure to altered feedback on people’s actions has a blurring effect on the perceived length of the arm, hand position or vividness feelings.

### Data Analyses

The data analyses followed the procedure described in Cardinali et al. (2009). For each experimental condition and for each of the 12 Pre- and Post-test trials, the mean velocity and the mean latency and amplitude of the peak velocity and acceleration of the index finger during the reaching movement were calculated. Movement onset was computed as the instant when the finger acceleration exceeded 0.5 m/s^2^ and movement end was computed as the instant when a peak in the forward direction of the finger position was detected, an event which corresponded with a negative peak in velocity and after which position stabilized (i.e., movement stopped). Movement time was calculated as the difference in time between movement onset and movement end. Then, an overall Pre-test value and Post-test value was calculated for each parameter based on the average of the twelve trials in the test. Based on the findings by Cardinali et al. (2009), longer latencies and reduced amplitudes in velocity and acceleration movement parameters from Pre- to Post-test would provide evidence of an internal representation of a longer forearm due to the audio-tactile tapping task. In addition, the reaching position served as an additional outcome measure: If the estimated length of the arm had increased by adaptation in response to the simulated sound source locations, participants were expected to reach toward a more proximal location, assuming that this movement would suffice to reach the remembered position (for related procedures see Lenggenhager et al., 2007; van der Hoort et al., 2011).

Behavioral trials were excluded from the analyses if the value of any of the parameters extracted for that trial shifted by more than two standard deviations from the overall mean for that given parameter. In total 1.7% of the trials were excluded from the analyses. As already mentioned, the trials in which participants failed to reach the target were not excluded from the analysis as they were important to calculate the effect over the average reached distances, which served as a measure of recalibration of the length of the represented arm. For all statistical tests, the alpha level was set at 5%.

## Results

### Reaching Task

The mean values ± SE for all measures are presented in **Table 1**. Initial analyses did not show any difference in the Pre-test values for any of the measures across Synchronous and Asynchronous conditions (*p* > 0.25), thus confirming the validity of these values as baseline.

**Table 1:** Results from the reaching task (*N* = 16).

Measure	Synchronous		Asynchronous	
		
	Pre-test	Post-test	Pre-test	Post-test
Mean velocity	810.63 (88.78)	713.68 (86.39)	785.32 (78.33)	741.42 (91.65)
Peak velocity	1625.68 (177.52)	1464.41 (179.81)	1578.58 (150.78)	1515.66 (186.66)
Peak acceleration	231.14 (53.47)	207.94 (47.40)	221.02 (42.46)	233.29 (57.20)
Latency peak velocity	204.86 (14.78)	227.43 (21.39)	204.28 (13.57)	216.16 (17.66)
Latency peak acceleration	50.99 (7.70)	61.35 (12.64)	47.87 (4.54)	51.05 (5.72)
Reached position	445.47 (3.49)	432.44 (3.47)	444.77 (3.27)	436.63 (4.33)
Movement time	595.23 (53.46)	693.57 (74.06)	647.51 (54.46)	717.39 (68.69)


*Mean velocity (mm/s), mean latency (ms) and amplitude of the peak velocity (mm/s) and acceleration (mm/s^2^) of the reaching movements, and mean reached position (in mm) and movement time (in ms) for each experimental condition and for both the Pre- and Post-test (all ±SEM).*

As in Cardinali et al. (2009), we split the participants into two groups (*N* = 8 in each group) according to their median arm length (70.5 cm) in order to check whether there were between-participant differences due to natural morphology (i.e., arm length in this case; see **Table 2**). A comparison of the mean Pre-test values (mean of the Synchronous and Asynchronous conditions) according to the ‘arm length group’ did not reveal a significant difference for any of the parameters (*p*-values for all parameters are: for mean velocity: *p* = 0.97; for peak velocity: *p* = 0.89; for peak acceleration: *p* = 0.83; for latency peak velocity: *p* = 0.51; for latency peak acceleration: *p* = 0.41; for reached position: *p* = 0.90; for movement time: *p* = 0.49). Nevertheless, similarly to the study by Cardinali et al. (2009) where differences were also non-significant, we could observe longer velocity latencies, longer movement times and smaller peak velocities in the ‘long arm’ group (Mean latency of peak velocity = 213.68 ms, *SE* = 20.29; Mean peak velocity = 1578.69 mm/s, *SE* = 279.29; Mean movement time = 656.52 ms, *SE* = 78.17) compared to the ‘short arm’ group (Mean latency of peak velocity = 195.56 ms, *SE* = 17.56; Mean peak velocity = 1625.56 mm/s, *SE* = 187.86; Mean movement time = 586.22 ms, *SE* = 59.85). This may suggest differences in kinematics of reaching movements between individuals with a longer arm and those with a shorter arm.

**Table 2:** Results from the reaching task split according to participants’ arm length (‘short arm’ and ‘long arm’ groups, *N* = 8 in each group).

		Synchronous		Asynchronous	
			
		Pre-test	Post-test	Pre-test	Post-test
Mean velocity	‘Short arm’ group	796.72 (105.90)	765.78 (109.80)	805.00 (97.39)	798.29 (118.61)
	‘Long arm’ group	824.54 (150.03)	661.58 (138.39)	765.63 (129.22)	684.54 (144.93)
Peak velocity	‘Short arm’ group	1613.50 (185.88)	1523.54 (172.03)	1637.63 (193.16)	1643.98 (223.92)
	‘Long arm’ group	1637.85 (316.95)	1405.29 (328.58)	1519.54 (243.17)	1387.34 (307.37)
Peak acceleration	‘Short arm’ group	201.95 (35.79)	211.95 (49.76)	229.60 (53.30)	245.55 (77.90)
	‘Long arm’ group	260.33 (103.58)	203.94 (84.57)	212.45 (69.77)	221.03 (88.94)
Latency peak velocity	‘Short arm’ group	198.64 (14.24)	217.89 (32.97)	192.48 (22.74)	204.16 (26.92)
	‘Long arm’ group	211.08 (26.89)	236.97 (29.13)	216.07 (15.23)	228.16 (23.90)
Latency peak acceleration	‘Short arm’ group	56.11 (12.35)	59.13 (16.24)	52.21 (6.78)	50.63 (6.15)
	‘Long arm’ group	45.8 (9.70)	63.5 (20.49)	43.54 (6.09)	51.47 (10.13)
Reached position	‘Short arm’ group	444.54 (4.63)	437.93 (5.10)	446.38 (5.70)	445.24 (5.45)
	‘Long arm’ group	446.40 (5.52)	426.96 (4.12)	443.15 (3.56)	428.02 (5.44)
Movement time	‘Short arm’ group	557.94 (81.43)	580.91 (98.87)	614.51 (71.23)	666.20 (101.01)
	‘Long arm’ group	632.52 (72.25)	806.24 (100.53)	680.52 (85.60)	768.57 (96.30)


*Mean velocity (mm/s), mean latency (ms) and amplitude of the peak velocity (mm/s) and acceleration (mm/s^2^) of the reaching movements, and mean reached position (in mm) for each experimental condition and for both the Pre- and Post-test (all ±SEM).*

Our main analysis focused on the effect of audio-tactile stimulation across Synchronous and Asynchronous conditions. For all variables we conducted Analyses of Variance (ANOVAs) with 2 × 2 within-subjects factors, ‘audio-tactile synchronicity’ (Synchronous and Asynchronous) and ‘time of test’ (Pre-test and Post-test), and the between-subjects factor ‘arm length group’ (long arm and short arm). We tested whether the distributions of the residual errors of the ANOVAs were normally distributed using the Shapiro–Wilk test. Only the residual errors related to the latency of the peak velocity (all *p*s > 0.194), the mean reached position (all *p*s > 0.233) and the movement time (all *p*s > 0.181) passed the normality test. Nevertheless, Q–Q plots for the residual errors of the variables mean velocity, peak velocity, peak acceleration, and latency of the peak acceleration showed moderate deviations from normality. Given that parametric statistical tests (ANOVAs) are quite robust to moderate deviations from normality (e.g., McDonald, 2014) we opted for the use of ANOVAs for all variables, which allow a factorial design and to explore the interaction between factors. Significant interactions were followed by planned pairwise two-tailed *t*-tests comparisons between Pre- and Post-test values for each audio-tactile synchronicity condition (with correction for multiple comparisons α = 0.025).

**FIGURE 2:**
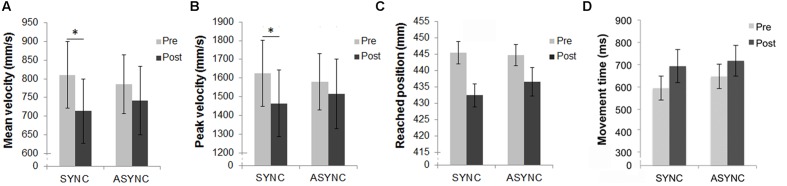
**Results from the reaching task.** **(A)** Mean velocity and **(B)** peak velocity of the index finger during the reaching movement, **(C)** mean reached position and **(D)** mean movement time, in pre- and post-test measures for each of the two experimental sessions (Synchronous and Asynchronous). Error bars indicate the SEM. ^∗^Denotes significant differences between conditions. In addition, the double interaction between ‘time of test’ and ‘audio-tactile synchronicity’ was significant both for the mean velocity and the peak velocity data.

**FIGURE 3:**
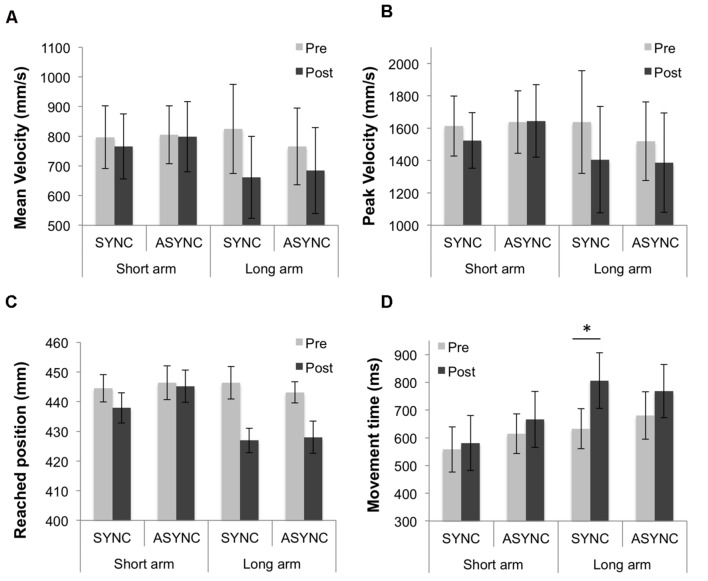
**Results from the reaching task split according to participants’ arm length (‘short arm’ and ‘long arm’ groups, *N* = 8 in each group).** **(A)** Mean velocity and **(B)** peak velocity of the index finger during the reaching movement, **(C)** mean reached position and **(D)** mean movement time, in pre- and post-test measures for each of the two experimental sessions (Synchronous and Asynchronous). Error bars indicate the SEM. ^∗^Denotes significant differences between conditions. In addition, the double interaction ‘time of test’ and ‘arm length group’ was significant for the mean velocity and the triple interaction between ‘time of test,’ ‘audio-tactile synchronicity’ and ‘arm length group’ was significant for the movement time.

For the mean reached positions, the main effect of ‘time of test’ [*F*(1,15) = 4.8; *p* = 0.045] was significant, while the effect of ‘audio-tactile synchronicity’ (*p* > 0.250) and the double interaction (*p* = 0.190) were not significant. Overall there was a significant decrease in the reached position from Pre- to Post-test, as displayed in **Figure 2C**, but this decrease did not significantly interact with the effect of audio-tactile synchronicity. There were no significant interactions between ‘arm length group’ and the within-subjects factors. Results split according to participants’ arm length are displayed in **Table 2** and **Figure 3C**.

For the mean velocity, the main effect of ‘time of test’ [*F*(1,14) = 11.54; *p* = 0.004] was significant, as well as its interaction with ‘audio-tactile synchronicity’ [*F*(1,14) = 5.36; *p* = 0.036], while the main effect of ‘audio-tactile synchronicity’ was not significant (*p* > 0.250). A significant decrease in the mean velocity from Pre- to Post-test was observed for the Synchronous condition [*t*(15) = 3.59, *p* = 0.003], but not for the Asynchronous condition (*p* = 0.118), as displayed in **Figure 2A**. Further, there was an interaction between ‘time of test’ and ‘arm length group’ [*F*(1,14) = 6.2; *p* = 0.026], due to a larger decrease in mean velocity from Pre- to Post-test for the ‘long arm’ but not for the ‘short arm’ group. This decrease reached significance only for the ‘long arm’ group [*t*(7) = 4.29, *p* = 0.004]. An inspection of the results summarized in **Table 2** and **Figure 3A** suggested larger Pre-Post differences in mean velocity in the critical (Synchronous) than in the control (Asynchronous) condition for the ‘long arm’ group than for the ‘short arm’ group (‘Long arm’ group: Mean Synchronous Pre-Post velocity change = -162.96 mm/s, *SE* = 32.07; Mean Asynchronous Pre-Post velocity change = -81.09 mm/s, *SE* = 33.24; ‘Short arm’ group: Mean Synchronous Pre-Post velocity change = -30.94 mm/s, *SE* = 29.08; Mean Asynchronous Pre-Post velocity change = -6.71 mm/s, *SE* = 38.84); however, the triple interaction between ‘time of test,’ ‘audio-tactile synchronicity’ and ‘arm length group’ did not reach significance (*p* > 0.23). These results suggest that the observed baseline shifts from Pre- to Post-test interacted with arm length and that this interaction was independent of ‘audio-tactile synchronicity.’

For the peak velocity, the main effect of ‘time of test’ [*F*(1,15) = 5.80; *p* = 0.029] was significant, as well as its interaction with ‘audio-tactile synchronicity’ [*F*(1,15) = 5.17; *p* = 0.038], while the main effect of ‘audio-tactile synchronicity’ was not significant (*p* > 0.250). A significant decrease in the peak velocity from Pre- to Post-test was observed for the Synchronous condition [*t*(15) = 3.34, *p* = 0.004], but not for the Asynchronous condition (*p* = 0.264), as displayed in **Figure 2B**. There were no significant interactions between ‘arm length group’ and the within-subjects factors (see **Table 2**; **Figure 3B**). Thus, our hypothesis that the auditory-induced illusory effect induced in the Synchronous condition modifies the kinematic of subsequent reaching movements was confirmed, providing evidence of changes in the represented arm length. For the peak acceleration, and the latencies, no significant effects, interactions or comparisons were found.

For the movement time (from movement onset to movement end), the main effect of ‘time of test’ [*F*(1,14) = 12.58; *p* = 0.003] was significant, while the main effect of ‘audio-tactile synchronicity’ or the interaction between both factors were not significant (both *p* > 0.21). A significant increase in the movement time was observed from Pre- to Post-test, as displayed in **Figure 2D**. Further, there was a triple interaction between ‘time of test’, ‘audio-tactile synchronicity’ and ‘arm length group’ [*F*(1,14) = 6.92; *p* = 0.020], due to a larger Pre-Post increase in movement time in the critical (Synchronous) condition for the ‘long arm’ group than for the ‘short arm’ group [*F*(15) = 10.91, *p* = 0.005], which was not observed for the control (Asynchronous) condition (*p* > 0.250; see **Figure 3D**). This decrease reached significance only for the ‘long arm’ group [*t*(7) = 4.84, *p* = 0.002]. These results suggest longer movement times from Pre- to Post-test; these baseline shifts interacted with arm length in the Synchronous condition, with longer movement times from Pre- to Post-test for the ‘long arm’ group.

Note that additional ANCOVA tests with 2 × 2 within-subjects factors, ‘audio-tactile synchronicity’ and ‘time of test’ (Pre-test and Post-test), and ‘arm length’ as a covariate, revealed a similar interaction on velocity data between ‘time of test’ and ‘arm length’ as the one reported for the mean velocity data analysis when ‘arm length group’ was treated as a between-subjects factors. The ANCOVAs showed a significant interaction between ‘time of test’ and ‘arm length’ for the mean velocity [*F*(1,14) = 15.12; *p* = 0.002] and for the peak velocity [*F*(1,14) = 9.45; *p* = 0.008], and a near significance interaction between ‘time of test’ and ‘arm length’ for the mean reached positions [*F*(1,14) = 4.10; *p* = 0.062]. For the peak acceleration, movement time, and latency data, no interactions were found.

### Questionnaire

**FIGURE 4:**
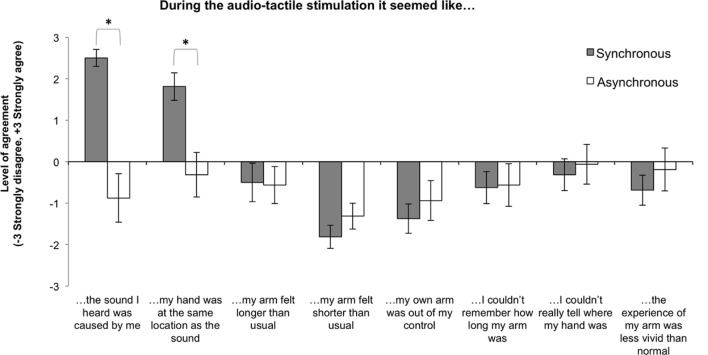
**Mean level of agreement for each questionnaire statement across Synchronous and Asynchronous conditions.** Error bars indicate the SEM. ^∗^Denotes significant differences between conditions as a result of the auditory manipulation.

The full set of statements and mean responses (±SEM) are presented in **Figure 4**. In order to investigate the effect of audio-tactile stimulation on the subjective experience of participants across the conditions, we used non-parametrical Wilcoxon Signed Ranks Tests to analyze the data. We observed significant differences between the two conditions for the first two statements. Firstly, while participants in the Synchronous audio-tactile condition felt that they caused the sound, they did not feel the same for the Asynchronous condition (*z* = 3.21, *p* = 0.001). Secondly, we also found that while participants in the Synchronous condition felt that the sound came from the same location where the hand was, they did not feel this happened in the Asynchronous condition (*z* = 2.64, *p* = 0.008). The disruption of feelings of being the agent of the sounds and of sound and hand being at the same location during the Asynchronous condition matches the results from previous studies and provides support to our choice of the Asynchronous condition as a control condition. Feelings of agency and of spatio-temporal congruency between action and sensory effect are necessary in order for auditory inputs to change body-representation (Tajadura-Jiménez et al., 2012, 2015b). Differences between conditions for the other statements did not reach significance (all *p*s > 0.1).

Then, given that the kinematics data revealed larger baseline shifts for the ‘long arm’ group in the Synchronous condition, which suggested larger illusory effects for this group, we also verified if the subjective experience of participants could be affected by arm length. We checked for possible individual differences in feelings elicited during the audio-tactile adaptation due to arm length. Based on the kinematic results, we hypothesized that, in the Synchronous condition, participants with longer arms would agree more with the statements related to a feeling of arm elongation, and potentially also with those statements related to a blurring of the perceived length of the arm or hand position. In order to investigate this hypothesis we conducted one-tailed Spearman’s rho correlations between the participant’s arm length and the self-reported level of agreement for all statements in the Synchronous and Asynchronous conditions. For the Synchronous condition we found that participants with longer arms agreed more with the statement *“my arm felt longer than usual”* [rS(16) = 0.45, *p* = 0.041] and *“I couldn’t really tell where my hand was”* [rS(16) = 0.47, *p* = 0.033]. All other correlations were far from the significance level.

### Correlations between Implicit and Explicit Measures

In order to investigate how the observed changes in our objective measures related to participants’ subjective experience, we performed correlation analyses between behavioral and subjective data. In particular, we conducted two-tailed Spearman’s rho correlations between the change from Pre- to Post-test in all kinematic parameters listed in **Table 1** in the Synchronous and Asynchronous conditions and the self-reported level of agreement for all statements in these conditions.

Results showed that, for the Synchronous condition, increases in mean reached position correlated significantly with increases in level of agreement with the statement *“It seemed like the sound I heard was caused by me”* [rS(16) = 0.52, *p* = 0.040], while decreases in mean and peak velocity correlated significantly with increases in level of agreement, respectively, with the statements *“my own arm was out of my control”* [rS(16) = -0.54, *p* = 0.03] and *“I couldn’t really tell where my hand was”* [rS(16) = -0.57, *p* = 0.022]. In addition, we observed that increases in the latency of the peak velocity correlated significantly with increases in level of agreement with the statement *“my arm felt longer than usual”* [rS(16) = 0.50, *p* = 0.047]. We also observed a near significant correlation between increases in movement time and the level of agreement with the statement *“my arm felt longer than usual”* [rS(16) = 0.49, *p* = 0.054].

We also found significant correlations between implicit and explicit measures for the Asynchronous condition, which proves agreement between measures. In this case, decreases in mean velocity and increases in movement time correlated significantly with increases in level of agreement with the statement *“my arm felt longer than usual”* [mean velocity: rS(16) = -0.50, *p* = 0.049; movement time: rS(16) = 0.56, *p* = 0.024] and that decreases in peak velocity correlated significantly with increases in level of agreement with the statement *“I couldn’t really tell where my hand was”* [rS(16) = -0.53, *p* = 0.036]; it should be noted, however, that participants overall disagreed more with these statements and that the behavioral measures indicated a smaller recalibration of represented arm in the Asynchronous condition than in the Synchronous condition. In addition, we observed that increases in the latency of the peak velocity correlated significantly with increases in level of agreement with the statements *“I couldn’t really tell where my hand was”* [rS(16) = 0.52, *p* = 0.038] and *“the experience of my arm was less vivid than normal”* [rS(16) = 0.54, *p* = 0.030].

## Discussion

The results from this study show that the manipulation of the spatial position of the sounds produced by one’s hand has an effect on the kinematics of goal directed arm actions. Importantly, this finding provides the first evidence of an auditory-driven recalibration of the internal models of body morphology that are possibly aimed at facilitating interactions with the environment. We show changes in the kinematics of reaching movements after periods of audio-tactile adaptation in which participants were exposed to spatially manipulated versions of the sounds generated by the tapping of their hand on a surface. These kinematic changes were characterized by reduced mean and peak amplitudes in the velocity of the reaching movements, and by longer movement times. Remarkably, these changes correspond with the kinematic profile of arm reaching movements performed by participants with longer arms [see Cardinali et al.’s (2009) study on tool-use for related results derived from extension in the represented length of the arm. See also their data, and ours, contrasting the kinematics of reaching movements of participants with long and short arms]. The observed kinematic changes when reaching toward a target relate to previous findings that one’s body is used as a “perceptual ruler” to measure objects’ sizes and distances and to accordingly guide bodily actions (Haggard and Jundi, 2009; Linkenauger et al., 2011, 2015; Canzoneri et al., 2013b; Keizer et al., 2013). Thus, changes in the represented size of the body result in a recalibration of the perceived size of the world (van der Hoort et al., 2011). Representing the arm as longer may lead to represent the distance to the target as shorter and in turn impact on the velocity of the reaching movement. In the following sections, we discuss these findings and their implications in further detail.

### Recalibration of the Internal Models of Arm Morphology Engaged in Action

We previously observed that exposure to the above-mentioned audio-tactile adaptation when tapping a surface with the hand may result in feelings of arm elongation and that it also changes the perception of tactile distances for objects in contact with the arm (Tajadura-Jiménez et al., 2012, 2015b). However, because multiple body-representations coexist in the human brain and their plasticity is a complex task-dependent process (Longo and Serino, 2012), the question of whether this adaptation to action sounds has an effect on the body-representations involved in facilitating action still remained open. The fact that in our previous studies we observed changes on tactile perception suggests a recalibration of somatosensory receptive fields (RF) in the primary somatosensory (SI) cortex. This recalibration has also been proposed in other works showing changes in tactile size perception induced by top-down sensory signals other than auditory ones (Taylor-Clarke et al., 2004; de Vignemont et al., 2005; Haggard et al., 2007; Canzoneri et al., 2013a,b; Miller et al., 2014). Given that the control of body movements performed when reaching for objects or manipulating tools relies on somatosensory representations of arm length (Holmes and Spence, 2004; Maravita and Iriki, 2004; Cardinali et al., 2009), we hypothesized that changes in arm motor behavior would follow after adaptation. The changes in arm motor behavior we observed in the current study provide support to our hypothesis of an auditory-driven somatosensory recalibration. Similarly, Cardinali et al. (2012) suggested that the changes in arm motor behavior following tool-use presumably followed a reorganization of SI RF geometry.

Previous experiments have reported that a general recalibration from Pre- to Post-test due to exposure to multisensory adaptation often occurs (e.g., Fujisaki et al., 2004; Vroomen et al., 2004; see also the results from our previous study, Tajadura-Jiménez et al., 2015b), and this general change was also observed in our results for the Synchronous and Asynchronous conditions. However, what is important here and in our previous study is that this recalibration interacted with the synchronicity of the stimulation during the adaptation phase. Here we found that the decrease in mean and peak velocity amplitudes from Pre- to Post-test reached significance only for the Synchronous condition. Further, for participants with longer arms we observed a larger Pre-Post increase in movement time in the Synchronous condition, which was not observed for the control (Asynchronous) condition. These results are in line with the findings from Cardinali et al. (2009) who took decreases in velocity and increases in the movement time as proxies of elongation in the represented arm. Noticeably, while we looked at seven kinematic parameters in arm reaching movements as a proxy of changes in the represented arm length, we did not find significant results for all the parameters. We found significant interaction between ‘audio-tactile synchronicity’ and ‘time of test’ for three parameters (mean velocity, peak velocity and movement time). Further, for the mean reached position we also found a main pre-post test change, and there was a trend toward an interaction between ‘time of test’ and ‘audio-tactile synchronicity.’ Indeed paired *t*-tests showed a significant decrease in reached position from pre- to post-test [*t*(15) = 2.68, *p* = 0.017] for the Synchronous condition, while this decrease was not significant for the Asynchronous condition. The ANOVA on the latency of the velocity did not reach significance, but we found that increases in the latency of the peak velocity correlated significantly with increases in level of agreement with the critical statement “*my arm felt longer than usual*,” as discussed in the next section, together with other correlations between changes in kinematics and in feelings. While we cannot explain why we did not find effects on the acceleration data, the effects we found in the velocity, latency, movement time and reached position, and the significant correlations between the kinematic data and the critical statements related to feelings of elongation or “blurring” of the hand location are in line with our hypothesis and also in line with the report from Cardinali et al. (2009). Note that in their experiments Cardinali et al. (2009) not always found effects in the same kinematic parameters and across experiments the number of parameters for which they found significant results varied (six, five, or four). A future study dedicated to investigate the effects in each parameter may include only one condition (Synchronous) allowing for a larger number of trials, which may result on significant effects in all parameters.

Alterations in arm kinematics as a result of changes in the somatosensory representation of arm morphology induced by action sounds may be interpreted in the context of ‘forward internal models’ of motor-to-sensory transformations (Wolpert and Ghahramani, 2000). These models are employed to predict movement dynamics, for instance, the position and velocity of one’s hand moving (Wolpert et al., 1995), as well as to do fine adjustments in the subsequent motor commands (Blakemore et al., 2002). The models are continuously updated during action execution, by using discrepancies between predictions and the actual sensory outputs that derive from one’s actions (i.e., reafference). Our previous results (Tajadura-Jiménez et al., 2012, 2015b) suggested that action sounds constitute part of this reafferent inflow and that the mental representation of the general body structure that allows the action to be produced (i.e., mental representation of the length of the arm) is updated in the process. However, before the current study there was no evidence of the effect of these updates on subsequent arm kinematics. A related study is one in which we show that increasing the frequency of self-produced walking sounds resulted in people estimating their body as being thinner and also in them changing their gait patterns in a way that was consistent with movements performed by a lighter walker (Tajadura-Jiménez et al., 2015a), but the current study is the first showing the effects of auditory-driven body-represented changes in goal-directed actions.

Taken together with our current results these findings suggest that action sounds contribute to the formation of body-representation and to guide bodily movements. It should be considered that these sounds are omnipresent since the auditory system provides a continuous stream of information (because our ears are not “turned off” in the same way that we regularly block vision by closing our eyes or by turning our head).

### Subjective Experiences in Response to the Manipulated Action Sounds

The results from the questionnaire replicated our previous findings that asynchrony between tapping action and sound disrupts the feelings of agency over the tapping sounds and of one’s hand being at the same location as the sounds (Tajadura-Jiménez et al., 2012, 2015b). Thus, results validate our choice of the Asynchronous condition as a control condition, as these feelings are necessary in order for auditory inputs to change body-representation (Tajadura-Jiménez et al., 2015b). Critically, the questionnaires did not show an effect on the feelings of arm elongation, neither for the Synchronous nor for the Asynchronous condition. Hence, while the observed kinematic changes suggest changes in the implicit representation of arm length that is needed to act and move (the often called body-schema; see for instance, Holmes and Spence, 2004 or Maravita and Iriki, 2004), these changes did not extend to the conscious representation of the appearance of the arm (the often called body-image). This finding is not entirely surprising, as such dissociations between implicit and explicit body-representation measures are often reported (e.g., Longo and Haggard, 2012). Similarly, in our previous studies looking at the effects of the same audio-tactile adaptation we did not find significant differences in the felt sensation of arm elongation when looking only at the questionnaire results (Tajadura-Jiménez et al., 2012, 2015b).

Nevertheless, in one of our previous studies using the same setup and paradigm described in the current study, we found a correlation between implicit (i.e., perceived tactile distance) and explicit measures of elongation in the represented arm (Tajadura-Jiménez et al., 2015b), which suggested similar processes taking place at the implicit and explicit levels. In the current study, we also found such correlation between implicit and explicit measures of elongation in the represented arm. For the Synchronous condition, we found that longer latencies of the peak velocity correlated with increases in the feeling that one’s arm is longer than usual. For this condition, we also found a near significant correlation between the feeling that one’s arm is longer than usual and longer reaching movement times. Note that in the study by Cardinali et al. (2009) the reaching movements performed after a period of tool-use were characterized by longer latencies and reduced amplitudes in velocity, and by longer movement times, and that the authors attributed these kinematic changes to a longer represented arm. Thus, the correlations we found in the Synchronous condition, are suggestive of elongation of the represented arm. A correlation between implicit and explicit measures of arm elongation was also existent in the control, Asynchronous condition. Previous studies on bodily illusions using synchronous and asynchronous multisensory stimulation conditions, such as those in the RHI, have reported that, overall, both synchronous and asynchronous stimulation elicit a range of ownership scores, but these scores are higher and more reliable for synchronous stimulation, and consequently, the Asynchronous condition is commonly used as a control condition (see discussion and references in Maister et al., 2013). Similarly, in our study the implicit measures indicated a smaller recalibration of represented arm and the explicit measures indicated lesser feelings of arm elongation in the Asynchronous than in the Synchronous condition.

Note that while we found that increases in mean reached position correlated significantly with increases in level of agreement with the statement *“It seemed like the sound I heard was caused by me,”* this positive correlation does not go against our hypothesis that a longer represented arm would lead to reach toward a more proximal location. Overall, feelings of agency in the Synchronous condition were always high, as reflected by the strong level of agreement with the statement *“I felt the sound I heard was caused by me,”* and there was an overall tendency to decrease the reached position from pre- to post-test. A tentative explanation for this correlation could be that, while agency is necessary to create the illusion, then this illusion may lead to surprise when reaching to the target (for instance, due to an unexpected delay in touching the table or to not having reached the target at all) and to an overall blurriness of the feelings related to one’s hand as captured in the self-reports collected after the experience. This blurring over one’s body was also reflected in the negative correlations found between another measure that we took as a proxy of the illusion of elongation in the represented arm (i.e., reduction in reaching velocity) and participants’ explicit reports of one’s arm being out of control and not knowing where one’s hand was. It is thus plausible that this blurring and loss of agency over one’s body and the sounds it produces are part of the process of updating body-representation, and it may be also possible that these feelings change during the time passed from the audio-tactile task and the subsequent reaching task to the moment when self-report is captured, and this is something that future research should clarify. Nevertheless, we can see that during the Synchronous condition participants strongly agreed with the statement *“I felt the sound I heard was caused by me,”* as already mentioned, and they also disagreed with the statements *“my own arm was out of my control”* and *“I couldn’t really tell where my hand was.”*

### Auditory-Induced Illusory Effects in Relation to the Actual Arm Length

We previously had found that the illusory effects on represented arm length when manipulating auditory sources depended on the distance to these sources (Tajadura-Jiménez et al., 2012). In particular, there were effects when the tapping sounds originated at double the distance to the tapping action but not at quadruple the distance, for which the illusion that the hand and the sound were at the same location broke (see Kilteni et al., 2012, for similar findings when manipulating visual sources). We interpreted our findings in the contexts of theories of ‘forward internal models’ of the motor system, in which it is assumed that larger temporal and spatial mismatches during the action-perception loop reduce the likelihood of forming an association between action and sound (Wolpert and Ghahramani, 2000; Ernst and Bülthoff, 2004). We hypothesized that auditory-induced illusory effects may be induced by auditory sources located in the “near space” but not in the “far space” of participants.

The “near space” is the region of space immediately surrounding the body, which in the field of cognitive neurosciences is sometimes also referred as “peripersonal space” (Lourenco et al., 2011). Various neuropsychological, neurophysiological and psychophysiological studies have evidenced that sensory information is processed differently for the space near and far from the body (Graziano et al., 1999; Farnè and Làdavas, 2002; Kitagawa and Spence, 2006; Tajadura-Jiménez et al., 2009). Some works have suggested that the specialization of brain areas in the processing of sensory events in the near space may be linked to a need for a larger sensorimotor control in this space (Graziano and Gross, 1998; Làdavas, 2002; Culham et al., 2006). At the same time, previous research has found individual differences in the distance of the boundary between one’s near and far space. In particular, the extent of the represented near space appears to scale with arm length, extending farther in the case of people with longer arms (Longo and Lourenco, 2007). Interestingly, studies on tool-use have shown that an elongation on the represented arm results also on an extension of the represented near space (peripersonal space), as evidenced by an extension of the area surrounding the body in which audio-tactile integrations are optimal (Canzoneri et al., 2013b).

While our study was not designed to test the hypothesis that auditory-induced illusory effects would be maximized when auditory sources are located in the “near space,” the recorded arm length allowed us to look at our results also in relation to this hypothesis. In particular, we hypothesized that the illusion would be larger for those people with longer arms as more sound sources would fall inside their near space and contribute to the illusion. The behavioral results were in support of this hypothesis: larger kinematics changes from the control Asynchronous to the Synchronous condition were observed for the ‘long arm’ group than for the ‘short arm’ group. The comparison between groups reached significance for the movement time parameter. This result suggests that people with longer arm experienced a larger illusion of arm elongation, as it is consistent with the reports of Cardinali et al. (2009) of longer movement time in free-hand reaching movements performed after tool-use (i.e., after elongation in the represented arm is induced). Note that Cardinali et al. (2009) also compared freehand grasping before tool-use with the first experience in using the tool and found that movement time was longer when grasping with the tool than with the hand. Further, the finding that the feeling of arm elongation reported after the Synchronous condition scales with arm length also suggests that auditory-induced illusory effects may be more easily induced by auditory sources located in the near space. Yet, a dedicated study should be conducted in order to provide further support to these hypotheses, which may include measuring the extent of the represented near space for each participant and relate it to the extent of the auditory-induced illusory effects. Also, a question that remains open is whether a manipulation suggestive of a shorter arm would produce opposite results to the ones reported here. It should be noted, however, that an illusion of body shrinkage might be more difficult to elicit than an illusion of body expansion, as there are much fewer reports in previous literature of the former than of the latter (Marino et al., 2010; although see for instance van der Hoort et al., 2011 or Banakou et al., 2013).

## Conclusion

The results presented in this study show that inducing in people a representation of an elongated arm, by altering the spatial position of the sounds generated by tapping their hand on a surface, makes them perform reaching movements in a way consistent with having a longer arm. These results provide the first evidence that body-representation changes induced by sound influence the kinematics of goal-directed actions. They provide further support to the hypothesis that the represented size of the body is used to calibrate the perceived distances to objects and to guide bodily actions. Further explorations may provide an insight on whether body-representation changes induced by sound are more easily induced when sound sources are located in the “near space” and on whether illusions of body shrinkage can be induced via sound.

## Author Contributions

All authors contributed to the conception and design of the work, interpretation of data and revision of the drafts of the work. AT-J acquired and analyzed the data, and drafted the work. All authors agreed to be accountable for all aspects of the work in ensuring that questions related to the accuracy or integrity of any part of the work are appropriately investigated and resolved, and approved this final version of the manuscript.

## Conflict of Interest Statement

The authors declare that the research was conducted in the absence of any commercial or financial relationships that could be construed as a potential conflict of interest.
