# Corrigendum: Action Sounds Modulate Arm Reaching Movements

**DOI:** 10.3389/fpsyg.2016.01878

**Published:** 2016-11-28

**Authors:** Ana Tajadura-Jiménez, Torsten Marquardt, David Swapp, Norimichi Kitagawa, Nadia Bianchi-Berthouze

**Affiliations:** ^1^UCL Interaction Centre, University College LondonLondon, UK; ^2^Department of Psychology, Universidad Loyola AndalucíaSeville, Spain; ^3^UCL Ear Institute, University College LondonLondon, UK; ^4^Department of Computer Science, University College LondonLondon, UK; ^5^NTT Communication Science Laboratories, NTT CorporationKanagawa, Japan

**Keywords:** auditory-dependent body-representation, action sounds, body-related sensory inputs, body kinematics, goal directed actions

Reason for Corrigendum:

In the original article, we forgot to add the following data access statement at the end of the Funding section: *All data created during this research are openly available from the UK Data Service ReShare archive at http://reshare.ukdataservice.ac.uk/852502.*

The authors apologize for this oversight. This error does not change the scientific conclusions of the article in any way.

## Funding

AT was supported by the ESRC grant ES/K001477/1 (“The hearing body”) and by the MINECO Ramón y Cajal research contract RYC-2014-15421. All data created during this research are openly available from the UK Data Service ReShare archive at http://reshare.ukdataservice.ac.uk/852502.

### Conflict of interest statement

The authors declare that the research was conducted in the absence of any commercial or financial relationships that could be construed as a potential conflict of interest.

